# The timing mega-study: comparing a range of experiment generators, both lab-based and online

**DOI:** 10.7717/peerj.9414

**Published:** 2020-07-20

**Authors:** David Bridges, Alain Pitiot, Michael R. MacAskill, Jonathan W. Peirce

**Affiliations:** 1 School of Psychology, University of Nottingham, Nottingham, UK; 2 Laboratory of Image and Data Analysis, Ilixa Ltd., London, UK; 3 Department of Medicine, University of Otago, Christchurch, New Zealand; 4 New Zealand Brain Research Institute, Christchurch, New Zealand

**Keywords:** Timing, Stimuli, Precision, Experiments, Software, Open-source, Reaction times, Sub-millisecond, MTurk, Online testing

## Abstract

Many researchers in the behavioral sciences depend on research software that presents stimuli, and records response times, with sub-millisecond precision. There are a large number of software packages with which to conduct these behavioral experiments and measure response times and performance of participants. Very little information is available, however, on what timing performance they achieve in practice. Here we report a wide-ranging study looking at the precision and accuracy of visual and auditory stimulus timing and response times, measured with a Black Box Toolkit. We compared a range of popular packages: PsychoPy, E-Prime®, NBS Presentation®, Psychophysics Toolbox, OpenSesame, Expyriment, Gorilla, jsPsych, Lab.js and Testable. Where possible, the packages were tested on Windows, macOS, and Ubuntu, and in a range of browsers for the online studies, to try to identify common patterns in performance. Among the *lab-based experiments*, Psychtoolbox, PsychoPy, Presentation and E-Prime provided the best timing, all with mean precision under 1 millisecond across the visual, audio and response measures. OpenSesame had slightly less precision across the board, but most notably in audio stimuli and Expyriment had rather poor precision. Across *operating systems*, the pattern was that precision was generally very slightly better under Ubuntu than Windows, and that macOS was the worst, at least for visual stimuli, for all packages. *Online studies* did not deliver the same level of precision as lab-based systems, with slightly more variability in all measurements. That said, PsychoPy and Gorilla, broadly the best performers, were achieving very close to millisecond precision on several browser/operating system combinations. For response times (measured using a high-performance button box), most of the packages achieved precision at least under 10 ms in all browsers, with PsychoPy achieving a precision under 3.5 ms in all. There was considerable variability between OS/browser combinations, especially in audio-visual synchrony which is the least precise aspect of the browser-based experiments. Nonetheless, the data indicate that online methods can be suitable for a wide range of studies, with due thought about the sources of variability that result. The results, from over 110,000 trials, highlight the wide range of timing qualities that can occur even in these dedicated software packages for the task. We stress the importance of scientists making their own timing validation measurements for their own stimuli and computer configuration.

## Introduction

Many scientists need high-precision timing of stimuli and responses in their behavioral experiments and rely on software packages to provide that precise timing. Indeed, we often hear people state that they use particular software packages because they “need sub-millisecond timing”. Yet there is a lack of information in the literature about what is actually possible to achieve with different packages and operating systems, and very few labs report testing the timing of their studies themselves.

Before going further, we should establish the distinction we draw between *accuracy* and *precision*. In general, precision is the more important issue for a behavioral scientist. Precision refers to the trial-to-trial variability of the measures: the jitter of the timing measurement or its “variable error”. Accuracy refers to the “constant error” of a measurement which, in timing terms, is often referred to as the “lag”, “offset” or “bias” from the true value. Accuracy issues commonly arise from hardware characteristics and represent physical limitations of the setup, like a stimulus at the bottom of the screen typically appearing several milliseconds after a stimulus at the top of the screen, due to pixels being rendered sequentially from top to bottom. If its magnitude is known, a constant offset in time (poor accuracy) can be corrected for by simply subtracting it from each measured value. Alternatively, in many studies the ultimate outcome measure is a difference between two or more conditions, and hence any constant error is canceled out by taking that difference. A variable error (poor precision) cannot be corrected afterwards, as by its nature, its value is not known on any given trial.

Here we compare the timing performance, as directly as possible, of several commonly-used behavioral science software packages, on various operating systems, and in both laboratory-based “native” systems and on studies conducted remotely via web-browsers. The aims were to (a) determine the range of timing performance that we encounter across platforms, packages and stimuli; (b) identify commonalities in performance issues that need to be considered; (c) assess whether online systems are technically capable of achieving sufficiently good timing for behavioral studies. We also hope the data will encourage users to test the timing performance of their own experiments directly, using measurement validation hardware. A study like this can only show what performance it is *possible* to achieve in a given setting rather than what is likely to occur in a standard experiment. Note, for instance, that we use a high-performance button box for our tests in order to minimize the timing errors caused by external factors (the keyboard). In contrast, many laboratory-based studies, and nearly all web-based studies, are run with a standard USB keyboard, which can add further latencies of 20–40 ms, depending on the keyboard (Neath et al., 2011).

There are also very few papers measuring *timing across packages*, allowing direct comparisons to be made based on similar hardware. Although comparisons across packages are no replacement for testing the timing on the system being used, the data presented in the current study do highlight a number of consistent effects that should be informative to users and might also provide an incentive for software authors to improve their packages. The only study we are aware of that compared timing across multiple software packages is that of Garaizar et al. (2014) and the follow-up paper (Garaizar & Vadillo, 2014) in which they corrected an initial error. That study compared DMDX (Forster & Forster, 2003), E-Prime (Psychology Software Tools, Pittsburgh, PA, USA) and PsychoPy but did so only on Windows 7, and only measured the precision of visual stimulus duration, without considering stimulus onset times, audio-visual asynchrony or response time measurements.

There also remains some confusion about the quality of *timing in online studies*, which are increasingly popular. As noted by other authors (see e.g., Reimers & Stewart, 2015), the rise in popularity is driven by the increasing ease with which participants can be collected by recruitment tools such as Amazon Mechanical Turk (or MTurk) or Prolific, and partly by improvements in web technology, especially in timing (Reimers & Stewart, 2015). To date, studies that have explicitly tested performance, using dedicated hardware to measure stimulus onset and generate responses at precisely known times, have aimed to test generic software technologies, such as the use of the JavaScript vs Flash, rather than comparing software packages that have been specifically written for the purpose of behavioral testing (such as jsPsych, Gorilla, or PsychoJS). These have shown that when used for stimulus presentation in web browsers, HTML5 has a slightly higher tendency to drop frames (Garaizar, Vadillo & López-de-Ipiña, 2014; Reimers & Stewart, 2015) than studies run in desktop (non-browser) software. Web technology is also currently improving at a dramatic rate; there have been a number of improvements since 2015 that suggest the need for newer measurements.

Measured reaction time errors in these online studies have been found to consist of a lag beyond the native applications of roughly 25–45 ms, depending on the system, and an inter-trial variability (standard deviation) of 5–10 ms (Neath et al., 2011, studies 5 and 6; Schubert et al., 2013, study 2; Reimers & Stewart, 2015, study 1). These studies did not compare any of the more recent online services such as Gorilla (Anwyl-Irvine et al., 2019), jsPsych (De Leeuw, 2015; De Leeuw & Motz, 2016), PsychoPy/PsychoJS (Peirce et al., 2019) or Lab.js (Henninger et al., 2019).

A very recent paper (Pronk et al., 2019) and another currently in pre-print (Anwyl-Irvine et al., 2020) have pointed to additional encouraging results in browser-based studies using touchscreens and keyboards. Pronk et al. (2019) used an Arduino-controlled solenoid to press on a range of touch-screen devices and keyboards and show response-time lags of 50–70 ms in most configurations (133 ms in one outlier) and inter-trial variability of 5–10 ms, depending on the browser. Anwyl-Irvine et al. (2020) report longer lags and greater variability, with timing errors occasionally in the hundreds of milliseconds and it isn’t clear what could have caused such errors. They did explicitly use modest-specification computers, avoiding machines with dedicated graphics cards, for instance, and using standard keyboards rather than high-performance button boxes, but those choices are in keeping with previous studies, including the most recent one by Pronk et al. (2019) and we would not expect them to cause errors of this magnitude. We note that they also used an outdated version of PsychoPy in their measurements and the timing of the online provisions improved a great deal in PsychoPy 2020.1. The aim of the current study was to isolate the performance of the software packages themselves, and also to be comparable with measurements of lab-based experiment software packages, so we opted to use a high-performance button box in all measurements, even though in web studies this would typically not be expected.

While some authors have consistently pointed out the need for higher precision and more testing of timing (Plant, Hammond & Turner, 2004; Plant & Turner, 2009; Plant & Quinlan, 2013; Plant, 2016), other authors have questioned whether sub-millisecond timing, of responses at least, is strictly necessary. Their point is that variability in response time measurements, once several trials have been averaged, should have relatively little impact on the statistical outcomes. For instance, Brand & Bradley (2012) modeled the effect of variability in a simulation study based on the known variability of participants and technical noise. They found, with a study of 158 simulated participants (in keeping with online studies), that the addition of “technical” noise to the simulated variability within participants made very little difference. Previous modeling work has also shown that timing errors, or at least lags, can be partially corrected with post-hoc calculations (Ulrich & Giray, 1989) although we aren’t aware of this being common practice.

Partly this result is due to the inherently high trial-by-trial variability in individual participants’ response times, which is on the order of several tens of milliseconds, depending on factors such as attention and motivation. Consider for instance the data from Reimers & Stewart (2007) where they compared response times (from real participants rather than a robot as in the current study), measured using code written in C vs Adobe/Macromedia Flash. Participants made a binary decision as fast as they could, which yielded a mean reaction time of between 375 ms and 400 ms (depending on the software setup) but the response times of individual trials had a range of over 300 ms (interquartile range of roughly 100 ms, SD of over 80 ms). That level of variability is almost certainly driven primarily by variance in human response times rather than in the software or hardware, which are generally of considerably smaller magnitude.

Furthermore, De Leeuw & Motz (2016) compared participants responding in a browser-based task with those in a lab-based version and found that, where there is a measurable difference in timing, this was predominantly in the form of an increased lag (decreased accuracy) but not increased variability. Given that many studies seek to measure effects based on a *difference in response* between conditions, it is only the variability that is usually a concern, as any constant lag is canceled out by taking a difference. That might largely explain the findings of Miller et al. (2018) who made measurements of various standard psychology effects, both online and offline, and found essentially no discernible difference in data quality between the online and lab-based data.

There are some forms of study, however, where sub-millisecond precision really is essential. For example, electroencephalography event-related potentials can have components that are very brief and more consistently timed than behavioral responses. Analysis of these can be dramatically impacted by a variability of only 10 ms in the trigger pulses with respect to the stimulus, or in the measured timing of the response. Even in behavioral tasks, in the absence of large numbers of trials or participants over which to average (unlike the ample 158 simulated participants in the Brand and Bradley study), high precision may be required.

Here, we quantify the technological variability (precision) and lag (accuracy) using dedicated testing hardware (Black Box Toolkit; Plant, Hammond & Turner, 2004), aiming to understand what precision can be achieved in ideal circumstances for a range of software. We suspect that the vast majority of studies will not achieve this level of precision due to, for instance, using a keyboard instead of a button box, or by not accounting for the display introducing timing errors.

We tested the fidelity with a range of timing measures that scientists often require. We measured *stimulus duration* to test whether a 200 ms stimulus really lasts for 200 ms. We measured the *stimulus onset*, relative to a TTL pulse (although this was not possible for browser-based studies) as would be needed to tag the stimulus onset with a trigger. Third, we measured the absolute timing of an *audio onset* relative to the same TTL pulse (in lab-based studies) and the *audiovisual synchrony* (in both lab-based and online studies). Lastly, we measured the *reaction time* to a visual stimulus using a robotic responder (the Black Box Toolkit key actuator), eliminating the physiological variability of human responses.

## Materials and Methods

We collected timing data for a range of common software packages in standard “native”, laboratory-based setups, for which we opted to use *PsychoPy* (v2020.1), *Psychophysics Toolbox* (v3.0.16 beta running on MATLAB R2018b), *OpenSesame* (v3.2.8) using *PsychoPy* backend (v1.85.3), *Expyriment* (v0.9.0), and NBS *Presentation* (v21 Build 006.06.19) and E-Prime (v3.0.3.8) run using E-Studio (v3.0.3.82).

We sought to compare the timing of those lab-based setups with several commonly-used online packages: PsychoPy/PsychoJS (v2020.1), Gorilla (Build 20190828), jsPsych (v6.0), lab.js (v2.4.4) and Testable. Testable does not give version numbers, but uses a rolling release. We recorded the Testable stimulus presentation data for all platforms on 23/10/19 and for response times on 07/08/19 for Linux 10/08/19 for Windows and 11/09/19 for Mac.

For the lab-based applications, a trigger pulse was generated by the software at the time at which it was intended for a visual stimulus to be displayed, or for an audio stimulus to start playing. The actual time of stimulus onset or offset was then measured via a hardware detector. We could therefore test absolute visual onset timing (compared to a hardware trigger from the package), absolute auditory timing (compared to the same trigger), visual duration precision for a 200 ms stimulus, audiovisual synchrony (attempting to present the two stimuli with simultaneous onset), and the measurement error of response time to a visual stimulus. For browser-based packages, this was not possible because web scripts do not have access to parallel or USB ports and so cannot generate a trigger signal, but all other measures were collected.

We created the experiments in the manner that might be expected from a normal user of each package (as described by the package documentation), and therefore excluded advanced, undocumented code additions to optimize performance. For example, the PsychoPy scripts were automatically generated by the graphical Builder interface, and were not supplemented with any custom-written Code Components to optimize performance. Since we are the authors of that package, we are more knowledgeable about potential optimizations than most users and it would be inappropriate for this package to receive any advantage from that additional experience.

As a caveat to the general rule of creating studies exactly as a typical user would, at times we weren’t sure what “typical users” would be aware of. For instance, in Presentation and in Expyriment, achieving a stimulus duration of 200 ms is certainly possible but to do so requires setting the requested duration to just under 200 ms (say, 195 ms). The timing mechanisms of those applications appear not to take into account the time to render the final frame, such that when requesting exactly 200 ms, the stimulus will actually overshoot by 1 screen refresh (typically 16.7 ms). While a naïve user *might not* take this into account, we considered it easy enough to apply that we should do so. Certainly, anyone validating the timing independently would notice the error, and be able to verify simple that the fix works in a reliable manner. Therefore, on those packages, we specified the stimulus duration to be slightly less than the intended duration.

### Equipment

*Linux and Windows* were tested on the same (dual boot) PC, with an AMD Ryzen 5 2600 6-core 3850 MHz central processing unit on a B450 Tomahawk motherboard, with 16 GB of DDR4 2133 MHz RAM, a Samsung 860 EVO 500 GB SATA III Solid State Disk, and a Gigabyte GeForce GTX 1050 Ti 4 GB graphics card. For *Windows* we used version 10 (10.0.18362), running the NVIDIA 417.01 graphics driver, and the REALTEK HD audio driver (6.0.1.8549). For *Linux*, we used the Ubuntu 18.04 operating system (Linux 5.0.0-31-generic), running the proprietary NVIDIA 430.26 graphics driver, with Advanced Linux Sound Architecture (ALSA) audio driver (1.1.3).

The *Apple Macintosh* hardware was a 2019 Mac Mini 64-bit 3.2 GHz Intel Core i7 with 16 GB of DDR4 2667 MHz RAM, with an integrated Intel Ultra High Definition (UHD) 630 1536 MB graphics processing unit. Testing was done on macOS X 10.14.5. The built-in Core Audio drivers were used for audio output.

The same monitor was used for presenting stimuli throughout: an AOC 238LM00023/I2490VXQ 23.8″ 60Hz LED Backlight LCD monitor with 1920 × 1080 pixel resolution and 4 ms response time (https://eu.aoc.com/en/monitors/i2490vxq-bt/specs). We confirmed that this model had no options to perform any ‘optimizations’ on the frames generated by the graphics card.

For online studies we used a range of browsers, as shown in Table 1.

**Table 1:** Browsers used for testing across the different operating systems. *Safari* and *Edge* are specific to *MacOS* and *Windows*, respectively. *Edge* was tested in two versions because Microsoft recently (with Edge version 78) changed the underlying engine to use *Chromium* (the open source engine behind the *Chrome* browser).

OS	64-bit Browsers				
	FireFox	Chrome	Safari	Edge	Edge Chromium
Mac	68.0.2	76.0.3809.1	12.1.1		
Win10	69.0.0	77.0.3865		44.18362.387.0	78.0.276.19
Linux	69.0.2	76.0.3809.1			

#### Measurement hardware

We used a Black Box Toolkit v2 (BBTK) to measure the onset and offset of trigger pulses, audio and visual stimuli. We also used it to trigger responses to the visual stimuli to test the response time measurements made by the software packages. Although that can be done by the BBTK all at once, in its Digital Stimulus Capture and Response (DSCAR) mode, that limited the number of trials we could include in a single run. We wanted to run 1,000 trials continuously and therefore opted to run the trials once to collect the trigger, visual and auditory onset/offsets, using BBTK’s Digital Stimulus Capture (DSC) mode, and then a second time using the response actuator to test the response timing of the software, in Digital Stimulus Response Echo (DSRE) mode.

Trigger (TTL) pulses were sent from all test systems using a LabHackers USB2TTL8 connected to the BBTK’s TTL 25-way ASC/TTL breakout board. A BBTK opto (photodiode), positioned at the center top of the display, was used to provide information about the visual stimulus. Audio onsets were recorded from the 3.5 mm speaker jack on the back of the computers.

#### Response time to visual stimuli timing

Responses to visual stimuli were created using the BBTK’s robotic response key actuator (RKA). The RKA was configured using the BBTK’s TTL 25-way ASC/TTL breakout board and the BBTK software RKA calibration tools in order to determine the onset and duration times of the RKA device. To achieve the desired onset times for the RKA, accounting for its solenoid response times, a 16 ms offset was taken from the intended response times.

The response actuator was positioned over button 1 of a LabHackers MilliKey, a 1 kHz USB response box that was used to collect responses on all platforms. Note that this is likely to provide a more precise measurement than in many lab scenarios, where standard consumer-grade computer keyboards are still commonly used. Only standard keyboards or touchscreens are used in nearly all online studies, but for comparison purposes, we considered it useful to measure a consistent high-precision response across all platforms.

### Procedure

Scripts for all the procedures (where the software provides a local copy to store) are available from Open Science Framework (https://osf.io/3kx7g/).

#### Response time latencies

For the response time measurements, we created an experiment in each package that simply presented a black screen for 300 ms, followed by a white square (positioned at the center top of the screen) for 200 ms. The experiment was programed to measure the response time of the actuator, which was programed through the BBTK to respond precisely 100 ms after the onset of the white square (triggered by the BBTK photodiode), with a keypress 50 ms in duration. This trial sequence was repeated 1,000 times in quick succession, following an initial pause of 5 s to give time for the BBTK to initialize in its DSRE mode.

#### Stimulus latencies

To measure the absolute and relative latencies of the visual and auditory stimuli, we programed an almost identical task that would present a similar black screen for 300 ms, followed simultaneously by the onset of a TTL pulse sent via the LabHackers USB2TTL8 trigger box, a white square at the top of the screen, and a simple audio tone, all lasting 200 ms. This simple trial sequence was again repeated 1,000 times for each package, following a 10 s initial blank screen while the BBTK initialized into DSC (Digital Stimulus Capture) mode. In some instances, using online software, it was necessary to present 1 trial of the auditory stimuli before main trials, in order to initialize the audio drivers and eliminate start-up delay in audio presentation for the first trial. If required, it is reported below for the relevant software.

To summarize, the differences between the *response time* runs and the *stimulus timing* runs are as follows. The serial port code for the TTL was only needed in the stimulus timing run (because the response timing was based on the visual stimulus it was unnecessary). The sound stimulus was also only needed in the stimulus timing run (again, it was unnecessary in the response timing run). Conversely, the keyboard checks were typically only needed in the response timing run and were omitted from the stimulus timing run.

Some aspects of the study implementations could not be kept exactly the same on all platforms. For instance, some packages *don’t* support mp3 audio files whereas others *only* support mp3 files. Some packages are able to generate their own white rectangle (as a Rect stimulus, for instance) whereas others required that to be loaded as a bitmap file. We doubt that any of these differences had any impact on timing. The stimuli were always loaded from disk at the start of the experiment, so the time taken to decode an mp3 file, or read an image from disk, should have no impact on the time taken to deliver the stimulus. Furthermore, all of these stimuli are really the most basic objects that we could imagine presenting and should not have impacted timing themselves.

Again, for full details of the experiments for all packages we provide the actual experiment files at Open Science Framework (https://osf.io/3kx7g/).

#### PsychoPy implementation

The aim was to mimic what relatively naïve users would normally do. To this end, the experiment in PsychoPy was created entirely in the Builder interface, except for Code Components used solely to automatically detect the LabHackers USB2TTL8 trigger box, set the status of the TTL object (e.g., started, stopped) and write triggers to the serial port in synchrony with the visual and audio stimuli presentation. The triggers were synchronized with the screen refresh using the PsychoPy Window method, callOnFlip(), which allows a call to be scheduled to run at the time of the next screen refresh, rather than immediately.

The black screens used throughout the task were generated by setting the experiment screen to black, in the Experiment Settings dialog. To generate the visual stimuli we used a Polygon component, a white rectangle 0.25 × 0.25 screen height units positioned at top and center of the screen. In the Experiment Settings, the audio library was set to be PTB (i.e., Psychtoolbox’s PsychPortAudio engine, ported to Python) with audio latency mode set to Level 3 (“Aggressive low-latency”). The sound waveform was generated by PsychoPy (i.e., an “A” tone was requested in the Sound Component settings, rather than a “wav” file being loaded). On macOS, a lower audio latency mode (level 1: “Share low latency driver”) was required to achieve clean sound (i.e., without crackling) on this Mac Mini, although that has not been the case on other Mac hardware that we have tested.

Components of PsychoPy experiments, in this case the visual and auditory stimuli and trigger pulses, can be run simultaneously simply by setting them to start and stop at the same time (whereas some of the packages only allow stimuli to be displayed sequentially). PsychoPy also has a check box for non-visual components to determine whether they should be synchronized with the visual stimulus (i.e., starting and stopping at the same time as the screen refresh, rather than as soon as possible) and this was set to be *on* for the Keyboard and Sound Components.

In general, the measurement and control in stimulus and response timing was achieved using frame refresh periods, where timing was set using a fixed number of screen refreshes, or frames, at the default refresh rate of 60 Hz (16.7 ms per frame). Thus, stimulus and response time latencies were converted into frame onset and duration using (*time in ms*/*frame duration*), for example the 300 ms visual stimulus onset was set to appear 18 frames from the beginning of each trial (300 ms/16.7 = 18 frames)—note, frames are rounded to nearest whole number. The exception was the sound duration. This was set to occur at a time in seconds because PsychoPy does not a provide setting for sound to be timed by frames, although it was also set to synchronize its onset with the screen refresh (the “sync visual” setting).

#### Psychtoolbox implementation

For Psychtoolbox there are multiple techniques one *might* use to control the stimulus timing and sequencing and so this is probably the package with greatest scope for users to get different timing than that described here.

In our implementation we timed the visual stimulus by actively drawing a fixed number of frames (rather than, say, flipping a frame to the display just once and then waiting for a fixed period until the next scheduled stimulus change). Many studies use dynamic stimuli that need to be updated continuously (i.e., on every screen refresh interval). That requires this active drawing and timing mechanism rather than a flip-once-and-wait method, and this was also a close match to the method used in the PsychoPy script. The trigger was sent by calling fprintf(usb2ttl, ‘WRITE 255\n’); immediately after the flip of the first frame. For frame refresh periods of all stimulus and response timing, see the PsychoPy implementation described above.

For stimulus timing, the sound stimulus was queued up before the first flip of the visual stimulus, by determining the time of the next screen flip using the PredictVisualOnsetForTime() function. This was then used as the when argument for PsychPortAudio(‘Start’,…). As with the PsychoPy implementation, the sound library was set to “aggressive low-latency” audio mode (level 3). Further, to play the sound synchronously with the visual stimulus rendering loop, we set the values of waitForEndOfPlayback and blockUntilStopped to be zero (off).

Response tasks used PTB’s PsychHID event-based functions (kbQueue and related functions) to keep track of key responses in parallel to stimulus presentation. At the end of each response trial, the keyboard buffer was emptied, and response times collected. These RTs were then added to a response matrix, and written to a text file at the end of the task. As PTB has no built in escape function, the stimulus timing task used a state-based keyboard polling method (kbCheck) to check for escape keys.

#### NBS presentation implementation

Presentation is designed for sequential presentation of visual stimuli, but does allow for parallel presentation of audio and visual stimuli.

In the Port menu, the output port was given an “Init sequence” of “WRITE 0\n”, a “Code sequence” of “WRITE 255 200000 0\n” and an “End sequence” of “WRITE 0\n”. The “Init” and “End” sequences are called at the beginning and end of the task. The “Code” sequence is called every time a “Code” parameter is specified in the task script. This “Code” string sent the instruction to the LabHackers device to set the TTL pulse ON for 200 ms, and set it OFF (zero) at the end of this sequence. Port device properties for the USB2TTL8 interface were set using Rate (155200), Parity (Even) Data bits (8), Stop Bits (1), clear-to-send, data-set-ready out/In set to ON, and data-terminal-ready and request-to-send were set to “enabled”. Also, the “FIFO Interrupt” checkbox was deselected.

In the Response menu, we added a keyboard device with the “1” button activated (the button used on the LabHackers Millikey response box). In the Video menu, the primary display driver was selected. In the Audio menu, we used the primary sound driver. The Presentation Mixer Settings were set to the low latency “exclusive” mode, according to NBS Presentation Audio Mixer Recommendations, with duplicate channels on load selected.

Both the response and stimuli timing tasks were coded in the Presentation script editor. A blank screen was generated using a blank text object, positioned in a picture object. To generate the visual stimulus we used a polygon graphic object, which defined a white 400 × 400 pixel rectangle, which was positioned at top and center of the screen, using a picture object. For the stimulus timing, an audio stimulus was created using a “.wav” file object, used to load a 200 ms long 440 Hz *wav* file, with the preload parameter set to true. The *wav* file object was added to a sound object, ready for presentation.

For both tasks, the trial timeline was generated using the trial object. In the trial object, we used the trial_duration variable to set the trial duration to 500 ms. For the response task, setting trial_type as “first_response” ended the trial on the first recorded response. The stimulus_event objects were used to present each of the following events. A blank screen starting from time zero for a duration of 300 ms, followed by a white stimulus, starting at 300 ms from zero, for a duration of 200 ms. In the stimulus timing task, trial duration was set to 500 ms, and the audio stimulus was presented at 300 ms, for a duration of 200 ms. For corrected onsets and durations, see the PCL code explanation below. The TTL trigger was added to the visual stimulus event only. To achieve parallel audio-visual stimulus presentation, we followed the Parallel Stimulus Events guidelines on the NBS Presentation website. This only required that we set the parallel parameter in the audio stimulus event to true.

PCL code was used to define the trial presentation, where the stimulus events were presented for 1,000 trials. Both tasks started and finished with a black screen for 1,000 ms. Note, we used Presentations black “ready” screen (see Settings tab) to provide the Black Box Toolkit initialization time. The offset of the blank screen and visual stimulus, as well as the onset of the visual stimulus, were corrected so that blank offset and visual stimulus onset duration was shortened by half a screen refresh (i.e., 200—screen refresh/2). Also, the visual stimulus onset began half a screen refresh before its desired onset of 300 ms, and thus started before the onset of the sound was scheduled (i.e., 300—screen refresh/2).

#### E-Prime implementation

E-Prime is also inherently a sequential stimulus presenter but can achieve simultaneous audio-visual stimuli using the Slide object. E-Prime (version 3.0.3.80) is not compatible with version 1903 of Windows 10, as used in this study, causing E-Prime to report the "Display is too busy" runtime error or freeze. To work around this, we needed to turn off Windows 10 “Fullscreen optimizations”, as recommended in the E-Prime documentation (https://support.pstnet.com/hc/en-us/articles/360024774913-ERROR-Experiments-run-on-Windows-10-May-Update-1903-or-Windows-10-November-Update-1909-freeze-or-receive-a-display-is-too-busy-error-30679).

Both tasks (stimulus measurement and response measurement) used the default experiment settings, with the exception of the Devices settings, where we set the Display to a specific refresh rate of 60, giving minimum acceptable refresh rate of 59, and a maximum acceptable refresh rate of 61.

The overall layout of both tasks was controlled using a main Procedure object, which started and ended with an Inline script object for setting the TTL trigger to its OFF state, and a black TextDisplay screen that ended on a keypress—useful to await BBTK initialization. No Inline code was required for the response timing task. The LabHackers USB2TTL8 was set up as a serial device in the experiment properties, where information can be sent to the serial port using the Serial object for example, Serial.WriteString “WRITE 0\n” to set an OFF signal at the start of every trial, and Serial.WriteString “WRITE 255 200000 \n” to set an ON signal for 200 ms, simultaneously with the stimulus. For the main trials, we added a List to the main procedure. The List acted as the main loop, where we created 1,000 samples (trials) by setting 100 cycles of 10 samples per cycle, using a sequential selection. To the main loop, we added another Procedure object for setting the trial timeline.

For the stimulus timing task, the trial procedure began with an empty Slide object, set to a black background. We used default Duration/Input properties of the Slide object. The duration of the Slide was set to 300 ms and PreRelease was “same as duration”, where this PreRelease setting allows E-Prime to preload the following stimulus immediately, during the presentation of the current stimulus.

For the stimulus presentation, we used another Slide object. The Slide was given a white background, and a SoundOut component, playing a 440 Hz wav file. We used default Duration/Input properties of the Slide object, where duration was 200 ms and PreRelease was “same as duration”. The PreRelease setting allowed immediate processing of the TTL code, positioned after the stimulus Slide object in the trial procedure.

For the response timing task, we needed only two Slide objects. The trial was the same as the stimulus timing task, without the sound component in the second Slide object, and without the InLine script for setting the TTL trigger. In addition the stimulus Slide object had additional settings in the Device/Input properties. Specifically, a keyboard was added as a device, with any key allowable, a time limit the same as the duration of the stimulus, and an End Action of “Terminate”, to end the trial on a keypress.

#### Expyriment implementation

Expyriment is also structured around sequential presentation. In each task, the stimulus was preloaded during the ISI to prepare the stimuli for fast presentation, then presented using the flip-then-wait method. We shortened the requested visual stimulus duration to 195 ms, which reliably achieved an actual duration of 200 ms. For both visual and audio stimuli, the present() method was called using the default values, which took approximately 1 ms to process, according to the returned time from the present() method. On each trial, the TTL pulse was fired immediately after the initial call to present the visual stimulus on screen.

#### OpenSesame implementation

OpenSesame is also structured around sequential presentation. For stimulus timing, in order to present the audio stimulus synchronously with the visual stimulus and TTL pulse we tested a number of ordering combinations. The best timing was achieved by a configuration in which the audio stimulus was presented first, followed by the visual stimulus, both with a notional duration of zero, such that the next object began immediately. These were followed by the TTL pulse, and then a call to *sleep* for the duration of the stimulus, so that the stimuli remained on screen for the correct duration. This is essentially a flip-then-wait method of stimulus presentation. We shortened the requested *sleep* duration to 191 ms, to achieve an actual duration of 200 ms. The TTL pulse (coded using the Python *pySerial* module) and calls to sleep (a method of the OpenSesame experiment class) were coded using inline script, an OpenSesame component for inserting custom code into the experiment.

In the response task, each trial presented a blank screen for 300 ms, followed by visual stimulus positioned at the top center position of the screen. The visual stimulus duration of zero allowed the task to move immediately onto the keyboard component, so responses could be collected whilst the visual stimulus remained on screen. The keyboard was given a timeout duration of 200 ms, with an event type of “keypress”, controlling the type of response collected (i.e., key down vs key up). Following the keyboard component, a Logger component was used to record the response time.

#### PsychoPy online (PsychoJS) implementation

The same study as used in the lab-based implementation was used to generate the PsychoJS script, which was then pushed to Pavlovia.org for running (all of which is done automatically by PsychoPy).

Parts of the code that were not needed for, or compatible with, the online version of the study, such as the connection to the hardware triggers, are automatically skipped by PsychoPy Builder during JavaScript code generation. No further customizations to the experiment were required for the study to run in the browsers.

PsychoJS uses WebGL where possible (unlike most of the other JavaScript packages, as far as we know). In just one configuration that we tested—Firefox on Linux—WebGL was supported but needed to be explicitly enabled on the PC we used. This is because Mozilla blacklists certain GPUs based on driver numbers to ensure that WebGL does not crash the browser if running on insufficient hardware. To turn off this blacklisting we opened the settings of Firefox and set layers.acceleration.force-enabled to true. This ensured WebGL compatibility of FireFox on Linux. Until we had made this adjustment, a warning message was provided that prevented the experiment from starting.

#### Gorilla implementation

We created a Gorilla Project, with separate Tasks for stimulus timing tests and response timing tests. Each Task was added to its own Experiment, where Task nodes were positioned between the start and finish nodes. For both tasks, we created an initial and final blank black screen, each 5,000 ms duration. For stimulus timing, we added an audio tone to the start screen in order initialize the audio drivers, ready for the task, followed by the main trials presenting a blank screen for 300 ms, followed by a stimulus screen for 200 ms, containing separate Zones for synchronous image and audio content presentation. For response timing, the main trials consisted of a 300 ms black screen, followed by a stimulus screen for 200 ms, containing separate Zones for image content presentation and keyboard responses. The task was run using the “Preview Task” option, used for piloting the task.

#### jsPsych implementation

The jsPsych task was coded using pure JavaScript, which consists of creating a timeline (array) of elements using jsPsych plugins (i.e., JavaScript objects with jsPsych compatible parameters used for presenting stimuli, recording responses etc.) and passing the timeline array as a parameter to the init method of the jsPsych experiment object. The stimuli used in both tasks were requested to be preloaded via the preload parameter in the jsPsych init method. To code the experiments, we created the timeline array, and added the Pavlovia.org connection object to the array. Finally, the command was sent to Pavlovia to finish the task and save any data.

For the response time task, we began with a welcome screen containing text, using an “html-keyboard-response” object. Pressing any key would begin the experiment. For each trial, we presented a black image to using “image-keyboard-response” for 300 ms, with no key options given, rendering the keyboard ineffective. Then, a stimulus screen presented a white stimulus on black background, using “image-keyboard-response” for 200 ms, with all keys allowed as a valid response. A response ended the trial.

The stimulus timing task was identical to the response time task, with the exception of the audio stimulus. On the stimulus presentation screen, the audio stimulus was presented simultaneously with the image stimulus using the “audio-keyboard-response” plugin, where the audio is passed to the stimulus parameter, and the visual stimulus is presented via the “prompt” parameter.

#### Testable implementation

We were informed that Safari cannot handle fast audio stimulus presentation via Testable (personal correspondence). Therefore, we did not assess Testable for synchronous sound and visual stimuli presentation using Safari.

Testable experiments are created using a comma-separated values (CSV) file, or Excel spreadsheet, where each row contains stimulus presentation configurations for each trial. For both response and stimuli timing tasks, tasks started and finished with a 5 s black screen. For response times, each main trial consisted of an ISI of 300 ms, followed by the 200 ms presentation of visual stimuli, with a keyboard response defined for each trial. For stimulus timing, a start-up trial was presented, preceding the 5 s start screen, containing an ISI of 300 ms, followed by the 200 ms presentation of audio and visual stimuli, in order to initialize the audio drivers, ready for the task. Each main trial consisted of an ISI of 300 ms, followed by the 200 ms presentation of audio and visual stimuli.

#### Lab.js implementation

The Lab.js task was built using the lab.js Builder. Both stimulus and response timing tasks had the same structure, and only differed with the addition of a sound oscillator for stimulus timing. The task started and ended with a black Canvas-based display, presented for durations of 5 s. We then added a Loop and set the sample to 1,000, where the sample denotes the number of loop iterations that will occur. To the Loop, we added a Frame component, which acts as the container for the stimuli. Frames contain the area occupied by the stimuli and only update that contained area on each screen refresh, thus potentially enhancing performance. This was advantageous with our stimuli since they occupied only a small portion of the screen. To the Frame, we added a Sequence, containing the trial events. To the Sequence, we added a black Canvas-based display presented for 300 ms for the ISI, and another Canvas-based display shown for 200 ms to present the stimuli. The stimulus canvas contained a white rectangle at the top-center location of the screen. The Behaviour of the stimulus canvas was set to have a timeout, or duration, of 200 ms, but was also able to record a key-down event, if required. For the stimulus timing task, sound was created using an Oscillator, added to the Behaviour timeline, with an onset at time 0, relative to the onset of the stimulus canvas, and a duration of 200 ms.

## Results

Note that we have deliberately not included significance testing on any of the measures presented here. Such tests would give a false impression of the results. The reason to provide tests would be to give a sense for the reader of whether this would generalize to their own hardware and environment but that is not something we can address. We have tested a large number of trials on a single machine and the variance we measured in that single machine is likely to be small compared with the variance between machines. We would therefore dramatically overestimate the significance of the differences with reference to the reader’s own configuration.

To create the tables here we have calculated a mean precision score for each row (each combination of package, operating system and browser where appropriate) and sorted the table according to that mean precision.

All raw data files and analysis scripts are available from Open Science Framework (https://osf.io/3kx7g/).

### Lab-based package results

**Figure 1:**
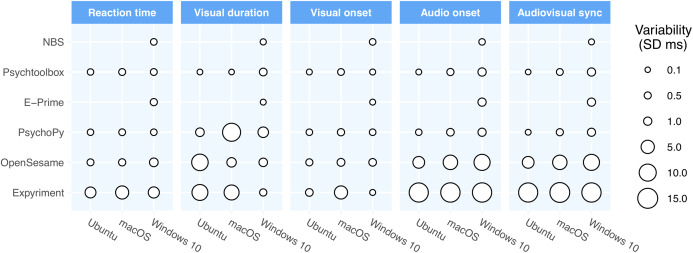
Precision across the packages and operating systems for lab-based software. The point size represents the standard deviation of the respective times in that configuration. In general, the majority of the differences are caused by differences between the packages (rows) although there are clearly some differences also between operating systems.

Table 2 shows the timing performance of packages running lab-based studies (not via a web browser) and Fig. 1 shows a visual representation of the variance in precision as a function of operating system and software package.

**Table 2:** Timing summaries of desktop software by package and platform. The Var(iability) measures are the inter-trial standard deviations of the various latencies for that configuration. The table is sorted by the mean of those variabilities (Mean precision). The Lag/Bias measures are the mean latencies for that configuration. In the case of audiovisual synchrony, a negative bias indicates the audio led the visual stimulus, a positive bias means the visual led the audio. Cells are colored pale green where times are “good” (arbitrary cut-offs of <=1 ms for precision and <=5 ms for lag). Cells are a dark pink where the timing is notably “bad” (>10 ms for precision, >20 ms for lag). An interactive version of the table can be found at https://psychopy.org/timing/2020/table2.html and this also provides links to plots of the data distributions.

Package	Platform	Mean precision (ms)	Reaction times		Visual durations		Visual onset		Audio onset		Audiovisual sync	
			Var (ms)	Lag (ms)	Var (ms)	Lag (ms)	Var (ms)	Lag (ms)	Var (ms)	Lag (ms)	Var (ms)	Lag (ms)
PsychToolBox	Ubuntu	0.18	0.31	12.30	0.15	2.05	0.18	4.53	0.17	−0.74	0.11	−5.27
Presentation	Win10	0.29	0.35	11.48	0.23	−1.83	0.34	7.07	0.31	0.56	0.19	−6.51
PsychToolBox	macOS	0.39	0.44	22.27	0.12	−2.15	0.41	21.52	0.53	0.09	0.43	−21.43
PsychoPy	Ubuntu	0.46	0.31	8.43	1.19	3.49	0.34	4.71	0.31	−0.71	0.16	−5.43
E-Prime	Win10	0.57	0.53	9.27	0.18	2.51	0.18	4.41	0.98	5.08	0.97	0.67
PsychToolBox	Win10	0.67	0.42	10.49	0.75	2.24	0.19	4.56	0.99	0.77	0.98	−3.79
PsychoPy	Win10	1.00	0.35	12.05	2.42	−1.97	0.35	7.10	0.96	0.85	0.93	−6.25
PsychoPy	macOS	2.75	0.40	22.02	11.56	1.00	0.55	18.24	0.70	0.54	0.52	−17.70
Open Sesame	macOS	3.14	0.54	21.21	1.65	18.94	0.79	18.10	6.40	9.46	6.30	−8.64
Open Sesame	Ubuntu	3.41	0.45	9.68	9.16	32.29	0.50	2.35	3.45	2.05	3.48	−0.30
Open Sesame	Win10	4.02	1.22	8.27	1.12	17.04	0.72	3.85	8.56	47.24	8.50	43.39
Expyriment	Win10	6.22	2.90	10.76	0.55	−0.08	0.19	5.98	13.72	106.83	13.72	100.85
Expyriment	Ubuntu	7.75	2.73	23.45	8.31	12.08	0.73	16.75	13.49	118.67	13.50	101.92
Expyriment	macOS	9.05	4.84	33.83	7.04	−1.13	4.82	29.02	13.84	42.81	14.72	13.79

Timing on the lab-based systems was generally impressive. Most of the packages tested were capable of sub-millisecond precision in the visual, audio and response timing tests used here. The packages typically show a constant apparent lag of roughly 4 ms in visual stimulus onset (visual onset bias), the difference between the occurrence of the trigger pulse and the pixels changing on the LCD screen. This lag is largely hardware-based and is position dependent—setting the stimulus lower in the display will result in a greater apparent lag. For PTB, Presentation and PsychoPy, which have settings to pre-schedule an audio onset there is also <1 ms lag for the audio stimuli.

For macOS, performance was less precise for visual presentations. This is due mostly to a known issue introduced in version 10.13 of macOS whereby a delay of 1 frame is imposed when updating the display. For most of the packages, this caused a relatively constant delay, and did lead to reduced *precision*. The resulting lag did, however, have a knock-on effect for other measurements, such as the visual response time measurement which then shows a lag of 20 ms on macOS for all packages. On Windows and Linux, the Windows Desktop Manager and the Linux Compositor, respectively, also have the potential to introduce presentation delays, but did not have an effect upon the data collected in this study.

*PsychoPy* performed well on all the timing tests under Windows 10 and Linux. Since version 3.2, PsychoPy has used the same engine as Psychtoolbox (ported to Python by Mario Kleiner), enabling excellent audio and response timing. It should be noted that earlier versions of the software did not attain this level of performance, so upgrading to PsychoPy 3.2+ is strongly recommended. As with the other packages, performance on macOS was poorer. In PsychoPy’s case, however, on this platform there appeared to be a reduced *precision* of visual stimulus presentation durations as well as the greater lag, which was not observed for the other packages.

*Psychophysics toolbox (PTB)* performance was excellent, at least on Linux and Windows. Achieving this precision does require more knowledge than when using PsychoPy’s automatically-generated scripts. That is, there are many ways to get poorer performance unwittingly but, when programed well, PTB can deliver excellent timing.

*E-Prime* performed very well out-of-the box, with no tweaking or effort. The audio stimulus had a slight (5 ms) lag compared to some of the other packages, but that is something that could presumably have been measured and corrected for, as was done for Presentation. Critically, the inter-trial variability (standard deviation) of the timings was sub-millisecond on every measure.

*NBS Presentation* timing was ultimately excellent, but this was not the case in the first instance. Initially we found duration measurements that overshot the desired 200 ms (which were corrected by requesting a duration of ½ frame less than the desired duration). We also initially found audio latencies to be both delayed and variable, having simply set the audio stimulus to play immediately after the visual stimulus. Detailed Presentation technical documentation on “Parallel Stimulus Events” describe a work-around that did allow the sound and visual stimulus to be prescheduled, if the user knows the latency that needs to be compensated for. Applying this compensation enabled the excellent timing shown in Table 2. To achieve this was rather more difficult than on other platforms, however, requiring familiarity with advanced documentation that many users will not have read.

*OpenSesame* timing performed well in the visual stimulus domain and response timing was also fairly good (an inter-trial variability of 1.16 ms on Windows was worse than the packages described above, but still adequate for most behavioral measurements). To get this response timing the package must constantly check the keyboard (it cannot do so asynchronously) which means that other stimulus updates can’t be made at the same time (whereas PsychoPy and PTB allow checking the keyboard while presenting dynamic stimuli) but, again, this would be sufficient for many simple tasks. Audio timing was less good, with a lag of over 40 ms and an inter-trial variability of 3–9 ms, depending on operating system. This poorer performance is because, at the time of writing, OpenSesame was using an older version of PsychoPy as its backend, which did not support the new PsychPortAudio library.

*Expyriment* had the worst performance in nearly all domains. Indeed, in many instances it was out-performed by the packages that were running experiments online. Expyriment’s stimulus presentation and response monitoring is built upon the Pygame Python library, which has not been optimized for low-latency, high-precision timing. We would not recommend the use of this package where precise stimulus/response timing is required.

### Web-based package results

In general, the *precision* of the web-based packages was reasonable, but *lags* were certainly more substantial than in desktop configurations. While these were constant within any one browser/operating system combination they varied a great deal between different combinations. There are also aspects of the timing, and especially stimulus onset lags, that could not be measured in online systems due to the lack of a trigger pulse from a parallel port (which cannot be controlled from JavaScript).

It is important to remember that the “online” response timing was measured as if the user had access to a low-latency button box. That is, we were using lab-based hardware from within a browser environment, which will not reflect the heterogeneous and generally low-spec commodity keyboards that will be used “in the wild” for online studies.

**Figure 2:**
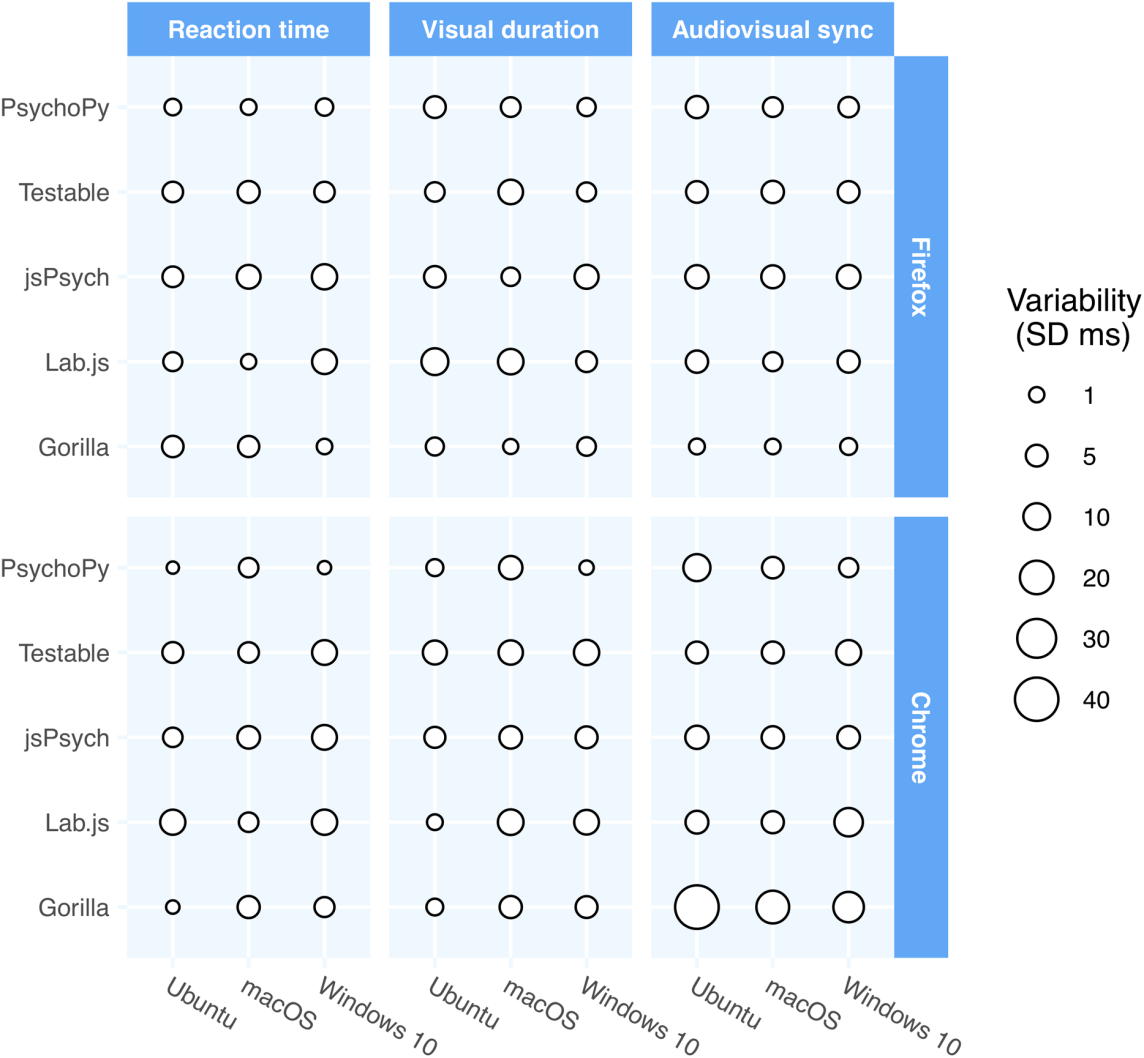
The precision across the packages, operating systems, and browsers for the two major cross-platform browsers. The point size represents the standard deviation of the respective times in that configuration. There is a greater mix of performance in the online provision, with some packages performing better on one browser/OS combination, and another package performing better on another.

Table 3 shows the performance of packages in browser-based studies and Fig. 2 shows a visual representation of the variance in precision as a function of operating system, browser and software package. Although the timing of the packages in online experiments did not match that of the lab-based packages, it was perhaps surprisingly good. The data were more mixed in terms of which packages performed the strongest, with some packages performing well on some browsers and poorly on others. Similarly, there was no clear winner in terms of operating system—Linux often performed poorly in these tests whereas it had generally been superior in the lab-based studies.

**Table 3:** Timing summaries of web-based software by package, platform, and browser. The Var(iability) measures are the inter-trial standard deviations of the various latencies for that configuration. The table is sorted by the mean of those variabilities (Mean Precision). The Lag/Bias measures are the mean latency values, for that configuration. In the case of audiovisual sync, a negative bias indicates the audio led the visual stimulus, a positive bias means the visual led the audio. Cells are colored pale green where times are “good” (arbitrary cut-offs of <=1 ms for precision and <=5 ms for lag). Cells are a dark pink where the timing is notably “bad” (>10 ms for precision, >20 ms for lag). An interactive version of the table can be found at https://psychopy.org/timing/2020/table3.html and this also provides links to plots of the data distributions.

Package	Platform	Browser	Mean Precision (ms)	Reaction times		Visual durations		Audiovisual sync	
				Var (ms)	Lag (ms)	Var (ms)	Lag (ms)	Var (ms)	Lag (ms)
PsychoPy	Win10	Chrome	1.36	0.39	43.95	0.67	−2.08	3.01	65.32
Gorilla	Win10	Firefox	1.84	1.11	24.83	2.67	1.35	1.73	88.27
Gorilla	macOS	Firefox	2.18	4.47	30.34	0.94	1.16	1.12	38.43
PsychoPy	Win10	Edge (Standard)	2.22	2.03	42	0.93	−2.28	3.69	56.19
PsychoPy	macOS	Firefox	2.65	1.17	67.01	3.38	0.24	3.4	−10.21
PsychoPy	macOS	Safari	2.66	1.05	33.5	4.26	0.49	n/a	n/a
PsychoPy	Win10	Firefox	2.76	1.96	40.97	2.42	−2.61	3.9	58.93
Gorilla	Ubuntu	Firefox	2.76	4.71	24.71	2.35	2.05	1.23	−30.61
jsPsych	macOS	Safari	3.39	0.66	31.31	4.39	3.09	5.11	−23.48
jsPsych	Win10	Edge (Chromium)	3.85	1.74	15.19	4.21	2.97	5.6	44.51
Testable	Win10	Firefox	3.92	3.87	31.36	2.94	1.91	4.95	76.32
PsychoPy	Ubuntu	Firefox	3.97	1.57	42.5	4.97	1.02	5.36	190.45
Testable	Ubuntu	Firefox	4.05	3.97	31.57	3.25	1.84	4.92	−44.36
PsychoPy	Ubuntu	Chrome	4.14	0.2	66.98	1.77	1.77	10.45	187.19
Lab.js	macOS	Firefox	4.2	0.97	16.38	8.61	19.51	3.01	4.26
PsychoPy	Win10	Edge (Chromium)	4.24	1.04	46.01	3.03	−3.36	8.66	63.3
jsPsych	Ubuntu	Chrome	4.63	3.23	48.29	4.33	4.05	6.34	27.73
PsychoPy	macOS	Chrome	4.84	3.22	35.29	6.47	−0.3	4.82	−6.86
Lab.js	Ubuntu	Chrome	5.12	8.27	31.79	1.19	2.34	5.91	198.05
jsPsych	Ubuntu	Firefox	5.12	4.11	31.38	4.84	4.05	6.43	10.55
jsPsych	macOS	Firefox	5.16	6.85	53.7	2.57	3.27	6.05	−15.29
Testable	Ubuntu	Chrome	5.46	4.23	47.09	7.06	−11.92	5.09	94.18
Testable	macOS	Chrome	5.52	3.99	41.94	7.43	6.34	5.13	67.2
jsPsych	macOS	Chrome	5.62	5.68	41.42	5.74	3.25	5.45	−9.71
Lab.js	Win10	Firefox	5.78	7.88	8.22	4.21	14.25	5.25	70.93
Lab.js	macOS	Chrome	5.79	3.26	20.3	8.78	6.51	5.31	−0.21
Gorilla	Win10	Edge (Standard)	5.96	4.95	40.23	7.15	4.95	5.79	70.93
Testable	macOS	Firefox	6.1	5.2	57.66	7.63	22.83	5.48	40.73
Lab.js	Ubuntu	Firefox	6.19	2.91	25.54	10.22	13.66	5.44	185.35
jsPsych	Win10	Chrome	6.23	7.85	23.27	5.1	3.6	5.73	43.57
Testable	Win10	Edge (Chromium)	6.8	4.11	15.99	8.34	−5.39	7.94	73.79
jsPsych	Win10	Firefox	7.38	8.37	25.7	7.04	15.32	6.74	32.32
Gorilla	Win10	Chrome	7.89	3.58	25.6	5.03	4.24	15.06	98.84
Testable	Win10	Chrome	8.08	7.88	23.96	8.38	−5.9	7.98	72.57
Lab.js	Win10	Edge (Chromium)	8.57	4.22	17.14	8.03	−4.9	13.45	82.45
Lab.js	Win10	Chrome	9.48	8.44	19.2	7.76	−1.73	12.25	86.03
Gorilla	macOS	Chrome	9.76	5.3	35.31	5.36	3.4	18.61	32.03
Gorilla	Win10	Edge (Chromium)	11.34	4.89	23.4	3.01	1.56	26.13	121.57
Gorilla	Ubuntu	Chrome	14.17	0.43	40.85	1.66	3.36	40.42	200.55
Gorilla	macOS	Safari	19.16	1.53	29.65	30.11	22.25	25.83	285.81

*PsychoPy/PsychoJS* version 2020.1 achieved an inter-trial variability under 5 ms in nearly all browsers for nearly all measures and often exceeded sub-millisecond precision. It should be noted that substantial timing improvements were made to this package in the 2020.1 release so users needing precise timing in their web experiments are strongly encouraged to upgrade and re-compile their JavaScript outputs from Builder.

PsychoPy had the lowest inter-trial variability in reaction times (under 4 ms on every browser/OS combination) with a mere 0.2 ms inter-trial variability for Ubuntu Chrome. Interestingly, the response time measure showed more *lag* under PsychoPy than some of the other packages, but better *precision*. We suspect that is due to PsychoPy using WebGL where available. That could well be introducing a 1-frame lag as the window is rendered, but then increases the certainty of when the rendering occurs. As discussed in this article, we consider constant lags of lesser importance than variability, so this may be an acceptable compromise, but we will certainly be trying to find ways using JavaScript to get low lags at the same time as low variability.

*Gorilla* performed relatively well in the visual tests, with consistently low variability across the browsers and operating systems. Similarly, it performed well with visual reaction times with under 6 ms inter-trial variability in all browsers and sub-millisecond in Chrome on Ubuntu. Where Gorilla struggled was with audio stimuli, with inter-trial variability over 10 ms in five of the browsers tested, and lags exceeding 100 ms in three cases.

*Lab.js* reaction time measures showed an inter-trial variability under 9 ms, with Firefox on macOS showing sub-millisecond precision. The notable thing about lab.js was that it showed surprisingly low *lag* values for measures like reaction time but not an improved precision. Indeed, on some trials, the lag was negative: it was reported as having a shorter response time than should have been possible, not something we saw in any other package or configuration.

*jsPsych* and *Testable* both showed inter-trial variability in the range 3.2–8.4 ms in all configurations, slightly less precise than Gorilla and PsychoPy. Nonetheless that variability is still less than the typical physiological variability of human participants themselves, or the keyboards and touchscreens that are typically used for responses.

## Discussion

From the data, it is clear that modern computers are capable of very precise timing in presenting audio and visual stimuli and in receiving responses, at least when used with a button box rather than a keyboard. There are a number of absolute lags that are common to all the software packages, which cannot be avoided, and there are differences between software packages but, in the best-performing packages on Windows and Linux, the inter-trial variability in the timing (the standard deviation of the measurements for a single configuration) was typically under a millisecond for all measures.

For lab-based studies, PsychoPy, Psychtoolbox, NBS Presentation and E-Prime were the most precise OpenSesame and Expyriment followed in precision, in that order. For online studies the timing was less precise than in the native applications and was quite variable between browsers. Stimulus duration remained relatively precise on most of the packages. For response times, there were larger lags, and these varied between browsers, but the precision within a software/browser combination (as would be experienced by an individual participant) was relatively good, with an inter-trial variability in the range 5–10 ms in most cases and even less for PsychoJS. The findings lend support to the notion that online studies might well be adequate across a large number of domains, except where the utmost precision is required, or where *absolute* response times must be compared between individuals, which would be impacted by responding on different systems. Further details on the particular packages and operating systems are considered below.

It is very important to note that the timings measured here represent something approaching a best-case scenario. Although we deliberately tested using mid-spec, rather than high-end, computers there are various reasons that our timing may have been better than in many standard experimental set ups. We used very simple stimuli: a single white square and a sound. We ensured that our monitors were correctly configured and the graphics settings were appropriate, whereas some labs, and most online participants, will leave the monitor and graphics card in whatever their default settings are. We also used a button box to measure the response times, rather than the standard commodity-grade keyboard used by many labs and nearly all online participants. Probably most importantly, however, by validating the timing independently with dedicated hardware, we could detect when timing was not as good as expected (whether because of hardware or software settings). For example, as mentioned above, we found that some of the software packages presented the visual stimulus for 1 extra frame (216.7 ms rather than the intended 200 ms) unless we reduced the requested duration to 195 ms. We suspect that a large number of studies are being conducted with timing that is considerably worse than reported here, by virtue of stimuli being incorrectly programed, hardware being incorrectly configured, or by computers that aren’t sufficient for the task. We discuss below a range of specific ways in which timing performance can be dramatically impaired. We would like to stress, once again, the importance of testing timing for every study, with the particular combination of stimuli, operating system and hardware used in that study. Papers such as this one, which report “best case” timing performance, should not be used in lieu of study-specific validation and testing.

### Comparing the lab-based packages

PsychoPy, Psychtoolbox, E-Prime and NBS Presentation all had precision that was below 1 ms on average across the measures. OpenSesame was slightly less precise across the board but most notably in audio. We were using the PsychoPy backend, but this in OpenSesame is currently using an older version of PsychoPy not supporting the new low-latency audio options. Expyriment had the worst timing and would not be recommended, particularly for studies needing precisely-timed audio stimuli. The exception in timing quality for lab-based studies was macOS, where all packages showed a large absolute lag in visual onsets, sometimes combined with a poor precision of those onsets, as discussed below.

For visual stimulus durations, most of the packages showed similar timing, although with Presentation and Expyriment, the correct duration was only achieved by setting the requested duration to a shorter time (setting to 200 ms resulted in a 1-frame overshoot, whereas setting it to 191 resulted in good timing). This is the sort of issue where, unless validating the timing with a hardware device, unsuspecting users would find it very easy to produce an incorrect stimulus duration. Surprisingly in OpenSesame we set the duration to be 191 ms but still observed an overshoot.

For response times, PsychoPy, Psychtoolbox, E-Prime and Presentation all provided reaction times with an inter-trial variability of 0.5 ms or less on all 3 operating systems. OpenSesame reaction times were slightly poorer on Windows, with an inter-trial variability of 1.2 ms, but similar high-precision on Linux and macOS. Expyriment reaction times had an inter-trial variability of 2–5 ms depending on the operating system. Due to the aforementioned visual lag on the Mac, the response times on that platform all appear to be 1 frame slower but, again, as that is roughly constant, the achieved precision is generally good.

The relatively poorer performance of Expyriment is likely to stem from its use of the Pygame library (a Python wrapper of SDL), which provides convenient features for programing, but sub-optimal performance.

### Comparing operating systems

Mario Kleiner, on the Psychtoolbox forum, has long advocated the use of Linux for optimal timing and that is somewhat born out here. Timing was indeed nearly always better on Ubuntu than the other systems, but the difference for these particular tests was relatively small (compare for example an audio variability of roughly 0.2 ms on Linux with 0.5 ms on macOS and 1.0 ms on Windows for both PsychoPy and Psychtoolbox). The difference may well be accentuated with tougher testing environments, such as testing whether the package still performs well under high computing loads.

The most notable poor performance overall was the lag of visual stimulus onset on Apple’s macOS. Attempting to sync a trigger pulse with a visual stimulus on a Mac revealed a 1-frame delay for most of the software packages, and on Expyriment the lag was longer and variable. This lag on the Mac is something that depends on the operating system version. Up to and including OS X 10.12, we could see the same high-precision visual timing as on the other operating systems but this changed in the system update from 10.13 onwards (persisting at least until macOS 10.14.5, as tested here). It appears that the system has added some additional buffering step (“triple buffering”) into its rendering pipeline. Therefore when the experimental software regards the framebuffer as having ‘flipped’, it has actually just progressed to the next buffering stage and is not yet visible on the screen.

The same behavior occurs in Windows 10 if triple-buffering is enabled in the driver settings, or by turning on screen scaling (which appears to implicitly use triple-buffering). On Windows these can always be turned off if the user knows to search for them. On macOS, however, there is currently no user-accessible way to disable this lag. In most other aspects, macOS had good timing.

### Comparing online packages

For the online packages, we could not measure the absolute lag as there is no means to send a hardware trigger synchronization pulse from within the browser environment. The only measures we could provide in that setting were the precision for a stimulus duration, the audio-visual onset, and the visual stimulus response. In many cases those are, in any case, the things that the scientist might need but they do little to inform us of the cause of any discrepancies. For example, when we find that the response time has a lag of 35 ms it isn’t clear whether this is caused by a delay in the visual stimulus appearing, or a delay in detecting the keypress.

Those caveats aside, behavioral scientists might be reassured to find that most of the online packages were certainly capable of presenting visual stimuli for a relatively precise number of milliseconds. For the vast majority of packages and browser/OS combinations, we found an inter-trial variability of less than 5 ms for stimulus duration and rarely any consistent under/overshoots. There was more variability than in the native packages, but the effects on most experiments are likely to be very small.

For response timing, similarly, we were generally impressed by the performance, finding the inter-trial variability to be under 10 ms in all cases. PsychoPy/PsychoJS topped this table recording a precision of under 4 ms in every browser/OS combination, and with sub-millisecond precision using Chrome for both Windows and Linux. As noted by other authors, the absolute lags in the response times were longer than in desktop studies, but these are typically the less important measure. In a study where one takes a measure by comparing the response times of participants across multiple conditions (consider a Stroop task, for instance, or an Implicit Association Test) then this is of little consequence. As we take the difference in response times, the absolute lag is subtracted and the only value of relevance is the variability.

Note that the measured timings here are much higher precision than in the most recent other study to our knowledge (Anwyl-Irvine et al., 2020). For instance, they report standard deviations of over 10 ms for most packages (Table 2), but that value is the standard deviation across devices and browsers for a package, not the inter-trial variability within any single configuration as reported here. A second key difference is that they used a keyboard for all measurements, although this would not be sufficient to explain some of the extreme reaction time measures that they found: for all packages they report maximum errors of over 150 ms, which we have not encountered in any of our measurements and which should not have resulted simply from the use of a keyboard. A last key difference with their data is that, for PsychoPy, Anwyl-Irvine et al. (2020) used an older version (v3.1.5) than in the current study (v2020.1) and it is certainly the case that PsychoPy’s timing has improved a great deal between those versions.

For audio-visual synchrony, the data are less encouraging; all the tested packages are currently struggling in that domain. There are some browsers where the synchrony is good, but others where it is extremely poor using the same software package. In some cases the sound failed to play reliably at all. The results indicate that JavaScript probably isn’t currently a technology ready for precisely-timed audio and this is an area for all the software authors to work on.

### The importance of making your own measurements

The comparisons made here generally represent best-case scenarios. There are several reasons that the timing would be poorer on a typical lab system than measured in the current study. The chief of these reasons is simply that we independently tested the timing with photodiodes and microphones and, in so doing, we found timing problems that could be addressed first. We consider below just some of the many factors that can cause timing quality to be reduced.

#### Visual lags from monitor and operating systems

The experimenter might experience lags of one or more refresh period for the visual stimulus (and that delay is effectively also added to your response times and to the audio-visual synchrony) in a manner that your stimulus presentation system cannot detect without dedicated hardware. This could be caused by your monitor itself. Monitors often now have multiple viewing “modes” such as movie mode or game mode, where the manufacturer seeks to optimize the picture for that particular activity. In optimizing the picture, they perform further processing on the pixel values that you have sent to the screen from your graphics card. As well as frustrating the careful scientist by subtly altering the images, the delay incurred can have a dramatic impact on timing, because the processing can take 10–20 ms, introducing a lag. Windows 10 can add a 1-frame lag if you turn on seemingly innocuous features like screen scaling. Unless you measure the physical timing against a hardware trigger signal, then you wouldn’t know that this was occurring. If you measure the system and establish that the timing is good but then stop measuring further, you are still susceptible to changes introduced as the operating system or the audio/graphics card drivers are updated (e.g., Apple silently introducing a 1-frame lag at the level of the operating system in macOS X 10.13). Note also that whereas some timing errors (such as a dropped frame) can be detected by the software packages themselves and will show up in log files and/or alert messages, others, such as image processing in the monitor, cannot be detected by any of the packages. Hardware tests are required to detect that if the monitor is adding delays to stimulus display.

#### Audio timing

Unlike the visual stimulus, for which we can at least detect when the graphics card has flipped a frame buffer (and then hope that the screen updated reasonably quickly after that), audio libraries do not report back on the progress they have made. When we request that a sound plays, we have no real information about when the speakers begin physically vibrating, except by recording it externally with a microphone. A software package might be able to use information about the size of the audio buffer and latency settings that are reported in the card to estimate when the sound is physically played, but it cannot detect any feedback signal that a sound has played. Again, the only way to know about the timing quality of your audio stimuli is to test with hardware devices.

#### Stimulus differences

The next reason to think that your timing performance might not match the performance described above is that, in nearly all cases, experimental stimuli will be more complex than those we presented here, and tests need to be carried out for *that stimulus* configuration to confirm that the quality of timing is still being maintained. Complexity can affect performance, and it might not be clear to many users what constitutes “complex” (for instance, rendering text stimuli is more computationally challenging for most packages than rendering a photographic image or a shape). Similarly, although we used computers with moderately high specifications rather than heavy-duty powerhouses, in reality very many experiments are run on relatively weak hardware. The characteristics of the computer and, especially its graphics card, can have a drastic effect on experimental timing. Especially in the days of high-resolution displays, many users are probably unaware of the demands that these place on a graphics card and that they should only be used in conjunction with high-end dedicated graphics processors. A “4K” display, increasingly common as a computer monitor, typically has a resolution of 3840 × 2160, which is roughly 8.3 million pixels, each consisting of red, green, blue and alpha values. At 60 frames per second the graphics card needs to update, and output, a staggering *40 billion values per second*. That is something that modern cards are capable of doing, but cheaper or older computers are not. When they encounter a high-resolution display, they will typically just send fewer screen updates per second, so that a nominally 60 frames per second display runs at, say, 30 Hz or becomes irregular.

#### Erroneous or inefficient code

While graphical experiment builders do a great deal to help reduce errors in code, it is still easy for a user to make mistakes. In particular, many users have rather little knowledge of the underlying limitations of their computer, or at least haven’t thought through the implications of those limitations. For example, it takes a relatively long time for an image to be loaded from disk and uploaded to the graphics card, whereas rescaling it and repositioning the same image has minimal overhead for a hardware-accelerated graphics application. At other times of course the scientist might have excellent knowledge but simply made a typographical error while creating the experiment. The best way to ensure that you haven’t made a mistake with your coding is to test the physical output of that coding (i.e., by testing the stimulus appearance with hardware).

#### Miscellaneous

There are many additional recommendations, of course. While running your study you should turn off unnecessary network services, such as Dropbox and email applications, as well as any applications that might be using substantial amounts of memory. You should keep image files to roughly the number of pixels that they will need when rendered on-screen (rather than loading a 12-megapixel image to be displayed on a 2-megapixel screen). You should try to perform any time-consuming activities in your code during inter-trial intervals or similar. If any of these miscellaneous issues are causing timing errors, however, most of them should be detectable in your software log files, which you should also check as a part of your testing process.

### How to test timing

There are a variety of options for this depending on your needs and budget. With an oscilloscope, a microphone, and a photodiode you can test audio-visual synchrony. If you don’t have a traditional oscilloscope there are now cheap PC-based options available to help, such as the BitScope (http://www.bitscope.com/), although those are more likely to require some programing (we haven’t tested one). The downside of traditional oscilloscopes is that while you can easily visualize the offset between various signals such as triggers and visual stimuli, this can be difficult or impossible to automate unless your scope also supports computer communication.

If you want to test the absolute lag of a visual or auditory stimulus then you will also need something with which to generate a trigger pulse, such as a parallel port. Physical parallel ports are increasingly hard to source, hard to configure and may not be compatible with your computer (e.g., on a Mac or a laptop PC). There are other cheap cross-platform USB solutions though, such as the LabHackers USB2TTL8 (http://www.labhackers.com/usb2ttl8.html) we used in this study and is extremely simple to set up and use, or a LabJack (https://labjack.com/), which has more channels and provides analog as well as digital in/outputs, but requires a little more programing effort.

The Labhackers Millikey (http://www.labhackers.com/millikey.html) is a high-performance button box with the additional feature that it can be commanded via the USB port (with RS232 protocol) to send a virtual keypress and this can be used to test how quickly the software then detected the keyboard event. We have tested this and found a round-trip time of under 1 ms to send the command to the box and detect the resulting keypress. An upgrade called the DeLux allows you to fire the virtual keypress on the basis of a visual stimulus onset, which you could also use to test how long it took to be detected by your software (after stimulus onset). Such a setup allows you to test the overall timing error from stimulus presentation to response collection (for instance, revealing the visual stimulus delay on recent macOS) and it can be scripted.

For the ultimate range of measurements, a key device is the Black Box Toolkit (BBTK, https://www.blackboxtoolkit.com/) (Plant, Hammond & Turner, 2004), as used in all the timing tests reported here. BBTK v2 has many inputs and outputs allowing you to test the timing over 4 photodiodes and 2 microphones and to send “responses” via sounders and keyboard actuators. You can use it to test the timing of stimuli, or to respond to your stimuli in numerous ways, all synchronized with trigger pulses over numerous TTL input/outputs. This is a more expensive device, costing between £1,500 and £3,000 depending on the options, but is highly recommended as a resource for complete testing of your experimental setup.

## Conclusions

We find that PsychoPy, Psychtoolbox, E-Prime® and NBS Presentation® are all capable of similar sub-millisecond precision in both stimulus and response timing. OpenSesame and Expyriment were slightly less precise, especially in terms of audio stimulus presentation. In comparing operating systems, the ideal system depends on the type of study: for visual stimuli Apple’s macOS suffers from a visual stimulus lag (since OS X version 10.13); Linux can be optimized to be extremely precise but its web browsers are seemingly poorly optimized, and Windows 10 appears to have reasonable performance in all domains.

For online studies we report the fastest, least variable data yet measured in browser-based latency tests. All the packages we tested were also reasonably precise in visual stimulus presentation, with PsychoPy achieving particularly impressive reaction time precision of under 4 ms on all browsers. That said, these packages remain not quite as precise as the lab-based equivalents. In particular, no online system can yet provide audio stimuli with precisely timed onsets. We also note, in agreement with previous studies (Reimers & Stewart, 2015), that the quality of *absolute* timing (the *accuracy* rather than the *precision*) is poor in browsers. Comparisons between participants, in terms of their absolute response times, is unwise for web-based data. Studies should aim always to make comparisons with a control condition on the same browser/computer (such as in within-subject comparisons) so that these absolute lags are naturally removed.

## Additional Information and Declarations

### Data Availability

The following information was supplied regarding data availability: All experiment code, raw data and analysis code are available at Open Science Framework: Peirce et al. (2020): “The timing mega-study: comparing a range of experiment generators, both lab-based and online”. OSF. Dataset. https://osf.io/3kx7g/.
